# Age‐related functional brain connectivity during audio–visual hand‐held tool recognition

**DOI:** 10.1002/brb3.1759

**Published:** 2020-07-18

**Authors:** Yanna Ren, Ao Guo, Zhihan Xu, Tao Wang, Rui Wu, Weiping Yang

**Affiliations:** ^1^ Department of Psychology College of Humanities and Management Guizhou University of Traditional Chinese Medicine Guiyang China; ^2^ Department of Psychology Faculty of Education Hubei University Wuhan China; ^3^ Department of Foreign Language Ningbo University of Technology Zhejiang China; ^4^ Department of Light and Chemical Engineering Guizhou Light Industry Technical College Guiyang China

**Keywords:** audio–visual integration, functional connectivity, hand‐held tool recognition, phase lag index, stimulus intensity

## Abstract

**Introduction:**

Previous studies have confirmed increased functional connectivity in elderly adults during processing of simple audio–visual stimuli; however, it is unclear whether elderly adults maximize their performance by strengthening their functional brain connectivity when processing dynamic audio–visual hand‐held tool stimuli. The present study aimed to explore this question using global functional connectivity.

**Methods:**

Twenty‐one healthy elderly adults and 21 healthy younger adults were recruited to conduct a dynamic hand‐held tool recognition task with high/low‐intensity stimuli.

**Results:**

Elderly adults exhibited higher areas under the curve for both the high‐intensity (3.5 versus. 2.7) and low‐intensity (3.0 versus. 1.2) stimuli, indicating a higher audio–visual integration ability, but a delayed and widened audio–visual integration window for elderly adults for both the high‐intensity (390 – 690 ms versus. 360 – 560 ms) and low‐intensity (460 – 690 ms versus. 430 – 500 ms) stimuli. Additionally, elderly adults exhibited higher theta‐band (all *p* < .01) but lower alpha‐, beta‐, and gamma‐band functional connectivity (all *p* < .05) than younger adults under both the high‐ and low‐intensity‐stimulus conditions when processing audio–visual stimuli, except for gamma‐band functional connectivity under the high‐intensity‐stimulus condition. Furthermore, higher theta‐ and alpha‐band functional connectivity were observed for the audio–visual stimuli than for the auditory and visual stimuli and under the high‐intensity‐stimulus condition than under the low‐intensity‐stimulus condition.

**Conclusion:**

The higher theta‐band functional connectivity in elderly adults was mainly due to higher attention allocation. The results further suggested that in the case of sensory processing, theta, alpha, beta, and gamma activity might participate in different stages of perception.

## Graphical abstract

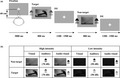

## 1 INTRODUCTION

In daily life, our brain can effectively screen and integrate effective information out of the dynamic complex information coming from the environment, and the process that merges information from various sense modalities (e.g., auditory, visual, olfactory, and somatosensory stimuli) is called multisensory integration (Laurienti, Burdette, Maldjian, & Wallace, 2006; Meredith, Nemitz, & Stein, 1987; Spence, 2011; Stein, 2012; Stein & Meredith, 1993). It is now well recognized that sensory decline is a normal part of the aging process (Grady, 2012; Mitchell, 2001), such as deficits in sound localization (Abel, Giguère, Consoli, & Papsin, 2000; Cui, O'Neill, & Paige, 2010), flash counting, (Setti, Burke, Burke, Kenny, & Newell, 2011; Stapleton, Setti, Doheny, Kenny, & Newell, 2014), temporal order judgement (Fiacconi, Harvey, Sekuler, & Bennett, 2013; Newell, 2012), speech perception (Babkoff & Fostick, 2017; Dey & Sommers, 2015), and object recognition (Pilz, Konar, Vuong, Bennett, & Sekuler, 2011); however, enhanced multisensory integration, particularly for audio–visual integration (AVI), was found for elderly adults compared with younger adults in auditory/visual discrimination tasks (Diederich, Colonius, & Schomburg, 2008; Peiffer, Mozolic, Hugenschmidt, & Laurienti, 2007; Zou, Chau, Ting, & Chan, 2017), sound‐induced flash illusion tasks (Deloss, Pierce, & Andersen, 2013), semantic discrimination tasks (Laurienti et al., 2006), and speech perception tasks (Sekiyama, Takahiro, & Shinichi, 2014), and it was further proposed that enhanced AVI might compensate for unisensory functional decline. Additionally, electrophysiological studies have also confirmed that elderly adults can enhance the activity of original audio–visual integrative brain regions (Diaconescu, Hasher, & McIntosh, 2013; Zou et al., 2017), recruit additional brain areas (Diaconescu et al., 2013; Ren, Ren, et al., 2018), or strengthen functional brain connectivity while completing some cognitive tasks, which indicated compensational mechanisms for the aging brain.

Functional connectivity is the mechanism for the coordination of activity between different neural assemblies to achieve a complex cognitive task or perceptual process (Fingelkurts, Fingelkurts, & Kähkönen, 2005), and it can be used to measure how well different brain regions cooperate during information processing. During cognitive processing, several oscillations are evoked simultaneously in a nonsynchronized way, and some of these frequencies can be enhanced by a resonance phenomenon when a stimulus is presented (Sakowitz, Quiroga, Schürmann, & Başar, 2005). To clarify the fundamental mechanism for the enhanced performance of elderly adults in audio–visual integration tasks, Wang et al. (2017) examined whether functional connectivity influences AVI during aging by an auditory/visual discrimination task. Their results showed that elderly adults activated stronger connections during audio–visual processing in the beta band than younger adults. Considering that AVI was affected greatly by the temporal relationship between the visual and auditory information, Wang et al. further investigated age‐related functional connectivity using audio–visual temporal asynchrony integration task (Wang et al., 2018). Similarly, stronger functional connectivity was induced in elderly adults, but in the theta band and alpha band, not in the beta band. They concluded that the higher functional connectivity might be due to greater cognitive demand in elderly adults. In the aforementioned studies, the simple meaningless audio–visual stimuli were used. However, the age‐related AVI studies have confirmed that the stimulus type influenced AVI greatly (Deloss et al., 2013; Diederich et al., 2008; Laurienti et al., 2006; Peiffer et al., 2007; Ren, Ren, et al., 2018; Wang et al., 2017, 2018; Zou et al., 2017). According to Wang et al.’s studies, the higher functional connectivity might be due to greater cognitive demand in elderly adults, however, in more complex and cognitive demanded task, whether there is aging effect in functional brain connectivity is unclear. Therefore, a particular interest of the current study was to clarify the age difference in functional connectivity observed during processing complex stimuli; and the dynamic hand‐held tool, containing biological motion and the presentation of a body part, was employed. Given that more cognitive recourse is needed during discriminating the dynamic hand‐held tool stimuli, the cognitive dysfunction in elderly adults, and the limitation of cognitive recourse for each person, we hypothesized that different functional connectivity oscillatory activities from the studies of Wang et al. are evoked.

As previous study, the neural oscillatory responses in the theta, alpha, beta, and gamma bands provide a potential mechanism for cross‐modal integration and information selection (Senkowski, Schneider, Foxe, & Engel, 2008). Theta oscillation has been suggested to be associated with attention (Keller, Payne, & Sekuler, 2017), working memory (Jensen & Lisman, 2005), and emotional arousal (Knyazev, 2007). Activity in the alpha band has been recognized as a marker of intentional ignoring and decreases with age, indicating deficits in the suppression of distracting signals (Friese et al., 2016; Keller et al., 2017; Yordanova, Kolev, & Başar, 1998), as well as working memory function (Başar, 2012; Keller et al., 2017; Palva & Palva, 2007). Given that attention decline and a suppressed distractor deficit in aging individuals have been extensively reported (Kok, 2000; Plude, Enns, & Brodeur, 1994; Quigley, Andersen, Schulze, Grunwald, & Müller, 2010), we hypothesized an increased theta‐band and reduced alpha‐band functional connectivity in elderly adults. Additionally, oscillatory beta activity is related to sensorimotor network processing and have further concluded a negative association with mean response times (Sakowitz et al., 2005; Senkowski, Molholm, Gomez‐Ramirez, & Foxe, 2006). Moreover, gamma‐band synchrony has been shown to be an elementary and fundamental process in whole‐brain operation (Başar, 2013). Aging effect studies have yielded evidence of dysfunction in executive function (Cepeda, Kramer, & Gonzalez de Sather, 2001; Kramer, Hahn, & Gopher, 1999), episodic memory (Loftus, 1984), and overall flexibility (Grady, 2012), and we hypothesized reduced beta‐ and gamma‐band functional connectivity in elderly adults.

Additionally, numerous studies have shown that AVI is greatly influenced by stimulus intensity, and the AVI is more pronounced for weak stimuli rather than strong stimuli when presented individually, which is called inverse effectiveness (IE) (Stein, 2012; Stein & Meredith, 1993; Yang et al., 2015). However, the IE is controversial in complex tasks, such as tool and speech recognition (Tye‐Murray, Sommers, Spehar, Myerson, & Hale, 2010). In their study, fifty‐three elderly adults and 53 younger adults instructed to conduct a closed‐set Build‐A‐Sentence (BAS) Test and the CUNY Sentence Test, and the low visual intensity was manipulated by degrading video contrast and low auditory intensity was manipulated by decreasing the signal‐to‐noise ratio, but no IE effect was found. However, the IE effect was found in the study of Stevenson et al. (Stevenson & James, 2009) using speech perception tasks. Therefore, another interest of the current study was to investigate whether the IE was evoked in the hand‐held tool recognition task by comparing the AVI effect and functional connectivity between high‐ and low‐intensity stimulus conditions. The IE was initially found in superior temporal sulcus (STS) (Stein & Meredith, 1993), and Stevenson et al. also reported that the STS displayed IE effect using functional magnetic resonance imaging. However, the behavioral response enhancement in Tye‐Murray et al.'s study and functional brain connectivity in the current study was used to evaluate the IE effect; therefore, we hypothesized that the IE effect was not occurred in the current study.

## 2 MATERIALS AND METHODS

### 2.1 Participants

Twenty‐one healthy elderly adults (57–70 years, mean age ± *SD*, 64.20 ± 3.02) and 21 healthy younger adults (19–26 years, mean age ± *SD*, 21.64 ± 2.54) were recruited to participate in the current study, and all the participants were paid for their time with RMB 50 per hour. All the younger adults were college students at Hubei University, and the elderly adults were citizens of Wuhan city. All participants were free of neurological diseases, had normal or corrected‐to‐normal vision, and were naive to the purpose of the experiment. Participants were excluded if their Mini‐Mental State Examination (MMSE) scores were >2.5 *SD*s from the mean for their age and education level (Bravo & Hébert, 1997). Additionally, the participants who reported a history of cognitive disorder were excluded from the experiment. All participants provided written informed consent for the procedure, which was previously approved by the ethics committee of Hubei University. Three elderly adults quit the experiment during the low‐intensity session because of physical fatigue, and one elderly adult was not able to control his head movements, which led to missing data. Therefore, seventeen elderly adults (57–69 years, mean age ± *SD*, 62.10 ± 2.90) and all 21 younger adults with normal cognition finished the whole experiment successfully and were used for further analysis.

### 2.2 Stimuli

**FIGURE 1:**
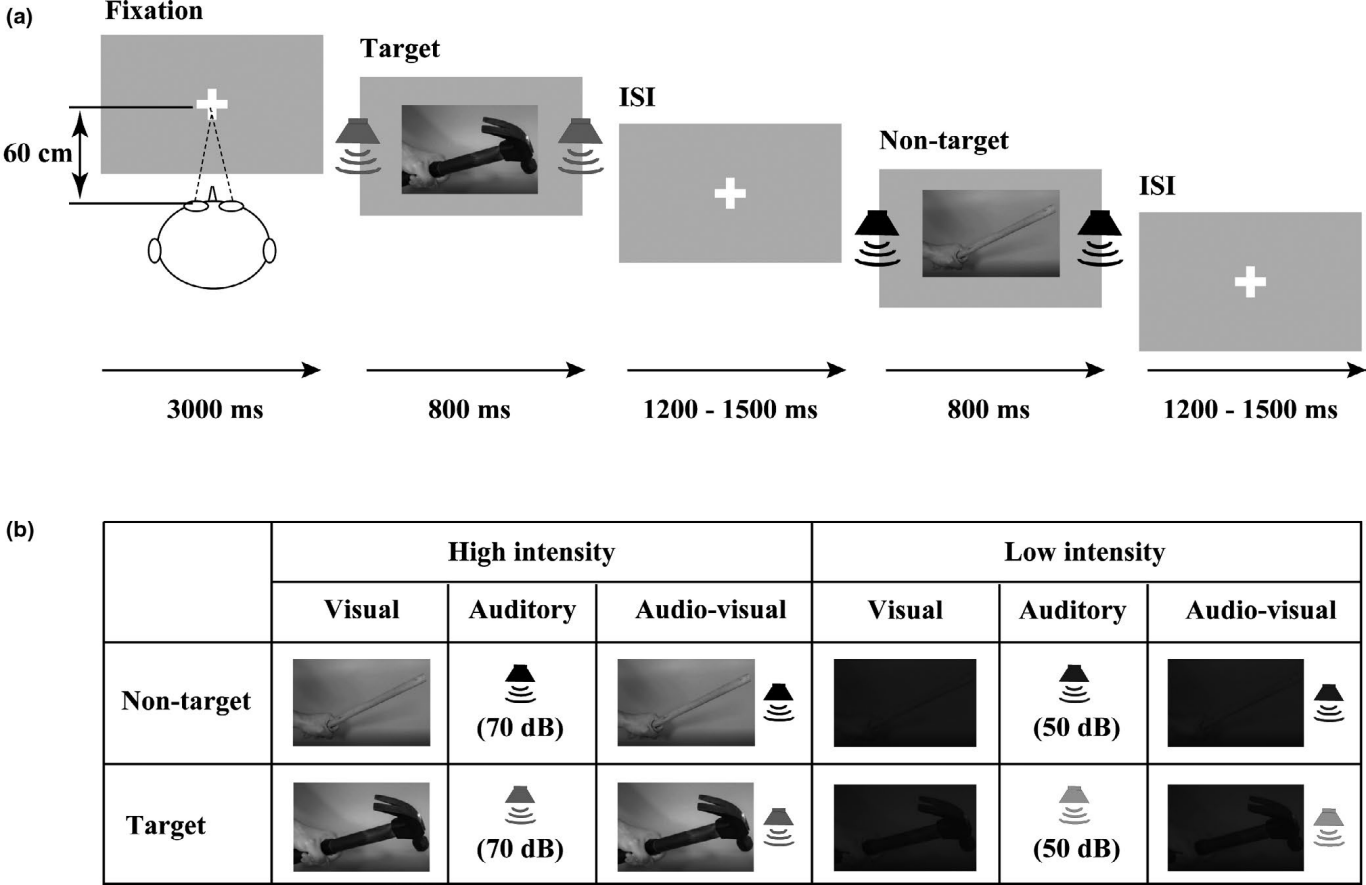
Schematic depiction of the experimental design. (a) An example of a possible sequence of the audio–visual target and audio–visual nontarget stimuli. (b) Types of stimuli

Stimuli consisted of 800‐ms digital audio–video recordings of a dynamic hand‐held tool (hammer and stick) and were recorded with a MiniDV Digital Camcorder (Sony; DCR‐PC55). To manipulate and present the visual and auditory stimuli separately, the individual video and audio files were extracted and processed from the raw recordings using Adobe Premiere CS6. The visual stimulus was the video acquired at the camera's original resolution of 966 × 544 pixels and converted from color to grayscale. The auditory stimulus was 32‐bit audio acquired at a sampling rate of 48 kHz with the camcorder's onboard microphone and was converted from stereo to mono. To ensure that the auditory and visual stimuli were comfortable for all participants, and the elderly and younger adults could reach to the similar hit rates for each stimulus under both high‐ and low‐stimulus‐intensity conditions, the intensity of auditory and visual stimuli was adjusted using double staircase method. Finally, in the formal experiment that the visual stimulus was the video down‐sampled to a resolution of 400 × 400 pixels, and the auditory stimulus was the original audio at 70 dB was applied in the high‐intensity session; and that the visual stimulus was the original with the luminance reduced by 70%, and the auditory stimulus was the original audio at 50 dB was applied in the low‐intensity sessions (Figure 1a).

The visual stimuli (V) were presented using a *Dell* computer monitor at a distance of approximately 60 cm from the participants (5.2 × 12.75 cm, with a vertical visual angle of 5° and a horizontal visual angle of 12°), and the auditory stimuli (A) were presented via speakers located on the left and right of the computer monitor (Figure 1a). The visual target was the video of a hand‐held hammer, and the auditory target was the audio of collision of the hammer against a marble floor. The audio–visual target (AV) was the simultaneous presentation of the visual target and auditory target. The visual nontarget stimulus was the video of a hand‐held stick, and the auditory nontarget was the audio of collision of the stick against a marble floor. The audio–visual nontarget was the simultaneous presentation of the visual nontarget and auditory nontarget stimuli (Figure 1b). The following conditions were not included: the video of a hand‐held hammer accompanied by audio of collision of the stick against a marble floor and the video of a hand‐held stick accompanied by the audio of collision of the hammer against a marble floor. During the experiment, the participant was instructed to press the right button if the target stimulus (A, V, AV) was presented as rapidly and accurately as possible, and was instructed to withhold their response when the standard stimulus (A, V, AV) was presented.

### 2.3 Procedure

The subjects were invited to participate the experiment on the workday from 17th Match to 27th May in 2019 randomly, and were instructed to perform the hand‐held tool recognition task in a dimly lit, electrically shielded, and sound‐attenuated room (laboratory room, Hubei University, China) with their head positioned on a chin rest. At the beginning of each session, the subjects were presented with a fixation cross for 3,000 ms, and then the target (A, V, AV) and nontarget (A, V, AV) stimuli were presented randomly for 800 ms (Figure 1a). Then a random interstimulus interval (ISI) of 1,200–1,500 ms was presented before the next stimulus. In total, 114 trials were conducted in each session, including 30 trials for each target stimulus type (A, V, AV) and eight trials for each nontarget stimulus type (A, V, AV). In total, eight sessions were conducted, including four high‐intensity‐stimulus sessions and four low‐intensity‐stimulus sessions, with each session lasting approximately 5 min. During the whole experiment, the high‐intensity‐stimulus sessions and low‐intensity‐stimulus sessions were performed in random order.

### 2.4 Data collection

The behavioral and EEG data were recorded simultaneously. The stimulus presentation and behavioral response were controlled using E‐prime 2.0. An EEG system (BrainAmp MR plus) was used to record EEG signals through 32 electrodes mounted on an electrode cap (Easy‐cap). Vertical eye movements and eye blinks were detected by deriving the EOG from an electrode placed approximately 1 cm below the subject's left eye. Horizontal eye movements were measured by deriving an EOG from one electrode placed approximately 1 cm from the outer canthi of the left eye. The impedance was maintained below 5 kΩ. The raw signals were digitized using a sample frequency of 250 Hz, and all the data were stored digitally for off‐line analysis.

### 2.5 Data analysis

#### 2.5.1 Behavioral data analysis

The response time (RT) faster than 200 ms or longer than 1,700 ms (due to either an omission error or long response) was excluded by the task program, and we did not exclude further outliers with any other method. The hit rate is the percentage of correct responses relative to the total number of target stimuli. The RTs and hit rates were computed separately for each subject under each condition. Then, the data was submitted to a 2_group_ (Elderly, Younger) × 2 _stimulus intensity_ (High, Low) × 3_stimulus type_ (A, V, AV) ANOVA (Greenhouse–Geisser corrections with corrected degrees of freedom). The statistical significance level was set at *p* ≤ .05, and the effect size (*η_p_^2^*) estimates are also reported.

To evaluate the AVI effect, the race model was used to analyze the behavioral data. The independent race model is a statistical prediction model based on the cumulative distribution functions (CDFs) of the summed probabilities of the visual and auditory responses to independent unimodal visual and auditory stimuli. This model allows the direct comparison of the probability of the multisensory condition to the predicted probability of the unimodal conditions [P(V) + P(A)] − P(V) × P(A)] by segmenting the subject‐specific CDFs for each condition using 10‐ms time bins (Miller, 1982, 1986). P(V) is the probability of responding within a given timeframe in a unimodal visual trial, and P(A) is the probability of responding within a given timeframe in a unimodal auditory trial. If the probability of the response to AV stimulus is significantly greater than that predicted by the race model (two‐tailed *t* test, *p* ≤ .05), integration of the auditory and visual inputs is considered to have occurred, and the time interval that AVI occurred is defined as time window of AVI (Diederich et al., 2008). The redundant nature of the audio–visual condition was defined by subtracting a subject's race model CDFs from his/her audio–visual CDFs in each time bin to generate a probability difference curve for each subject. For all subjects, the probability difference curves were average to obtain the mean probability difference curves. The greatest audio–visual facilitation of the mean probability difference curves is defined as peak benefit, and the time spanned from the presentation of the target to the maximal benefit is defined as the peak latency, which was used to assess the time point when AVI occurred together with the time window of AVI as in previous study (Diederich et al., 2008; Ren, Suzuki, et al., 2018; Ren, Yang, Nakahashi, Takahashi, & Wu, 2016). Besides, the individual peak latency was also obtained from probability difference curve, and the statistical analysis of significance between elderly and younger adults was conducted basing on the individual peak latency (two‐tailed *t test, p* ≤ .05). Additionally, the positive area under the curve (AUC) was calculated to comprehensively evaluate the AVI ability.

#### 2.5.2 EEG data analysis

##### Preprocessing

The EEG data were imported and processed with MATLAB R2013b (MathWorks, Inc.) with the open source EEGLAB toolboxes: EEGLAB (Swartz Center for Computational Neuroscience). The EEG data were positioned according to the 32‐channel montage of the international 10/20 system, and only those data elicited by the nontarget stimuli condition were analyzed to avoid motor effects. First, the two electrodes monitoring eye movement (horizontal EOG and vertical EOG) were deleted, and then the data were re‐referenced to the bilateral mastoid electrodes (TP9 and TP10). The remaining continuous EEG data were bandpass filtered from 1 to 50 Hz during recording at a sampling rate of 250 Hz, and the data were divided into epochs with 700 time points (800 ms prestimulus and 2,000 ms poststimulus points). Third, an independent component analysis (ICA) was used to remove artefacts (e.g., eye artefacts, frequency interference, muscle artefacts, head movement, and electrocardiographic activity) from the data (Delorme & Makeig, 2004; Jung et al., 2001; Makeig, Jung, Bell, Ghahremani, & Sejnowski, 1997), and all the channels were subjected to baseline correction. Finally, the A, V, and AV nontarget data were extracted independently for further analysis.

##### Functional connectivity

First, the instantaneous phase measures for each trial epoch and each electrode were calculated by employing the short‐time Fourier transform (STFT) using a windowed Fourier transform (WFT) with a fixed 200‐ms‐long sliding hamming window and 1‐Hz steps to obtain the power spectrum (Figures S1 and S2). According to the previous references and the analysis results in our laboratory, such a time–frequency analysis was chosen to achieve a good trade‐off between time resolution and frequency resolution in the range of theta‐, alpha‐, beta‐, and gamma‐band EEG frequencies (1–50 Hz) (Zhang, Peng, Zhang, & Hu, 2013; Zhang, Hu, Hung, Mouraux, & Iannetti, 2012). Second, the phase difference (Δφ) between two specified electrodes at a given time point and frequency (*t, f*) was calculated by an Phase lag index (PLI), as shown in (1) adapted from Cohen's study (Cohen, 2014), and then the PLI was used to assess functional connectivity between the two specified electrodes. Here, the n is single trial index, N is the total trial number, *t* is the time index, and the *f* is the frequency index.

(1) $$\text{PLI}\left(t,f\right)=\frac{1}{N}\sum_{n=1}^{N}\text{sign}\left(\Delta \varphi_{n}\left(t,f\right)\right)$$

Finally, the PLI for each stimulus (A, V, AV) was filtered into theta (4–7 Hz), alpha (8–13 Hz), beta (14–30 Hz), and gamma (31–50 Hz) frequency ranges and then averaged for each frequency range. The PLI analysis produces an electrode‐by‐electrode adjacency matrix (28 × 28) across trials (700 time points) for each participant. To avoid distortions and repetition involved in calculating the STFT at the edges of the analyzed epochs, the first 600 ms (150 sample points) and last 1,400 ms (350 sample points) were not displayed in the synchrony analyses (Wang et al., 2017, 2018). Thus, only the adjacency matrix for the reduced epochs (200 time points) from 200 ms before to 600 ms after stimulus onset was reported.

To investigate the difference of global functional connectivity between elderly and younger adults, the mean weight (PLI values) of all connectivities in the network was calculated across sensors for each condition and each subject as follows (Wang et al., 2017, 2018): First, the adjacency matrices (28 × 28) at each time point were averaged for each trial condition, and the average network connectivity time series was used to evaluate functional connectivity dynamics. Second, one‐tailed *t* tests were employed at each time point to compare the differences in the mean PLI values between the two groups to locate the time window of the age‐related differences, and the statistical significance level was set at *p* ≤ .05. A time point with a significant group difference indicates a difference in the global functional connectivity between the elderly and younger groups when processing stimuli. Third, to easily compare the differences in the group and the stimulus intensity, the PLI values in the time windows across significant time points were averaged for each condition, and then submitted to the 2_group_ (Elderly, Younger) × 2_stimulus intensity_ (High, Low) × 3_stimulus type_ (A, V, AV) ANOVA to estimate the diversity in the global functional connectivity during stimulus processing for elderly and younger adults under the high‐ and low‐intensity‐stimulus conditions. The statistical significance level was set at *p* ≤ .05, and the effect size (*η_p_* ^2^) estimates are also reported.

## 3 RESULTS

### 3.1 Behavioral performance

**FIGURE 2:**
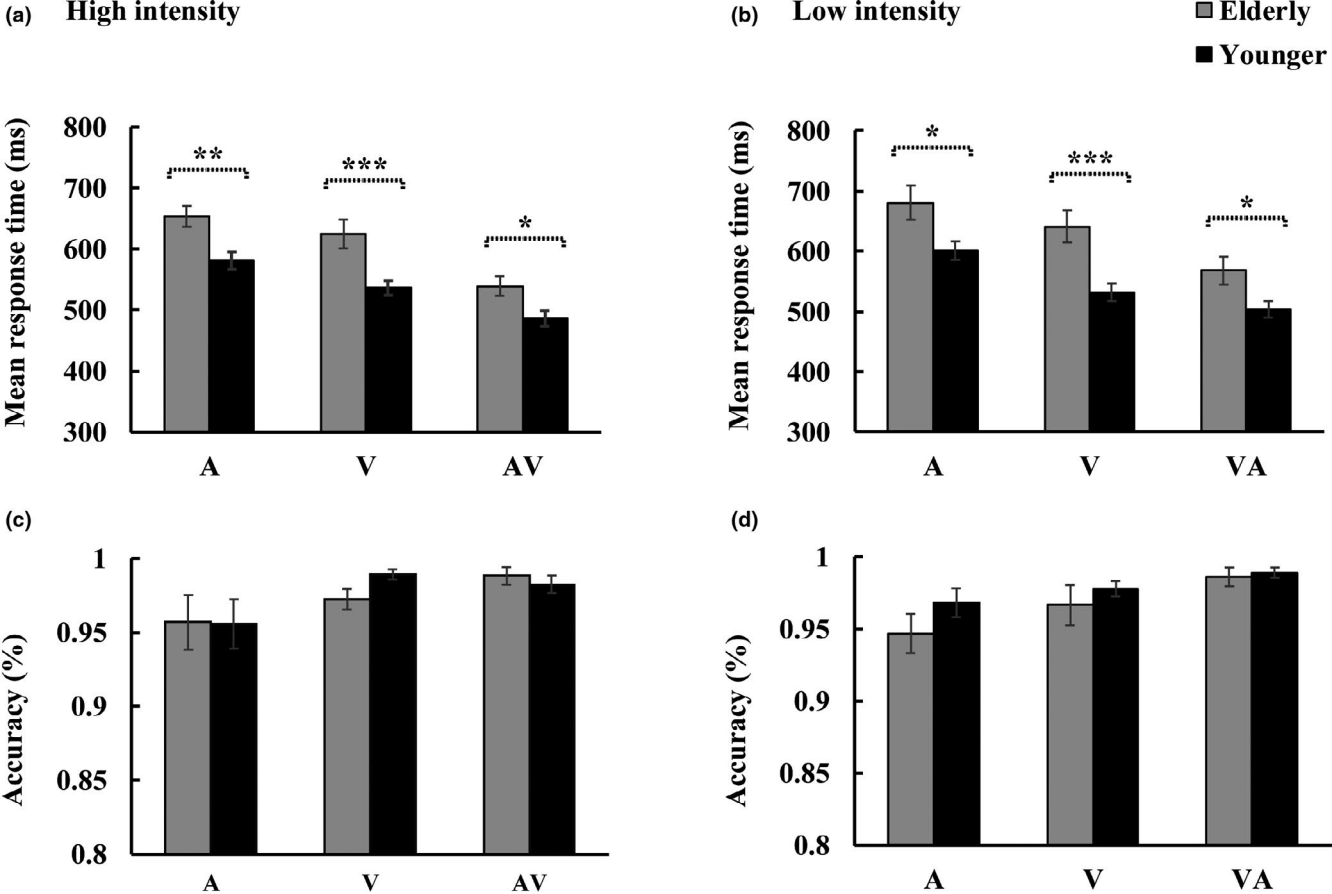
Mean response times and hit rates under each condition with standard error of mean (*SEM*). A significant difference in the response time was found between the elderly and younger adults under both (a) the high‐ and (b) the low‐intensity‐stimulus conditions. No significant difference in the hit rates was found between the elderly and younger adults under either (c) the high‐ or (d) the low‐intensity‐stimulus conditions. ^*^ *p* ≤ .05, ^**^ *p* ≤ .01, ^***^ *p* ≤ .001

The 2_group_ (Elderly, Younger) × 2_stimulus intensity_ (High, Low) × 3_stimulus type_ (A, V, AV) ANOVA for RTs showed significant main effects for group [*F*(1, 36) = 12.006, *p* = .001, *η*_p_^2^ = 0.250], showing a faster response to the target by the younger adults than by the elderly adults, and for stimulus intensity [*F*(1, 36) = 5.868, *p* = .021, *η*_p_^2^ = 0.140], showing a faster response to the target under the high‐intensity‐stimulus condition than under the low‐intensity‐stimulus condition (Figure 2a,b). Besides, a significant main effect for the stimulus type [*F*(2, 72) = 101.72, *p* < .001, *η*_p_^2^ = 0.739] was also found, showing a faster response to the audio–visual target than to the individual auditory and visual targets (AV > V > A). Additionally, a significant interaction between the group and stimulus type [*F*(2, 72) = 3.827, *p* = .028, *η*_p_^2^ = 0.096] was also found. The *post hoc* analysis showed that for all the stimulus types, the response to the target was faster in the younger adults than in the elderly adults (all *p* ≤ .01). For both the elderly and younger adults, the response to the audio–visual target was the fastest (AV > V > A, all *p* ≤ .016). The 2_group_ (Elderly, Younger) × 2 _stimulus intensity_ (High, Low) × 3_stimulus type_ (A, V, AV) ANOVA for the hit rates only showed a significant stimulus type main effect [*F*(2, 72) = 7.632, *p* = .002, *η*_p_^2^ = 0.175], showing higher hit rate for the audio–visual stimuli than for the individual auditory and visual stimuli (AV > A > V; Figure 2c,d).

**FIGURE 3:**
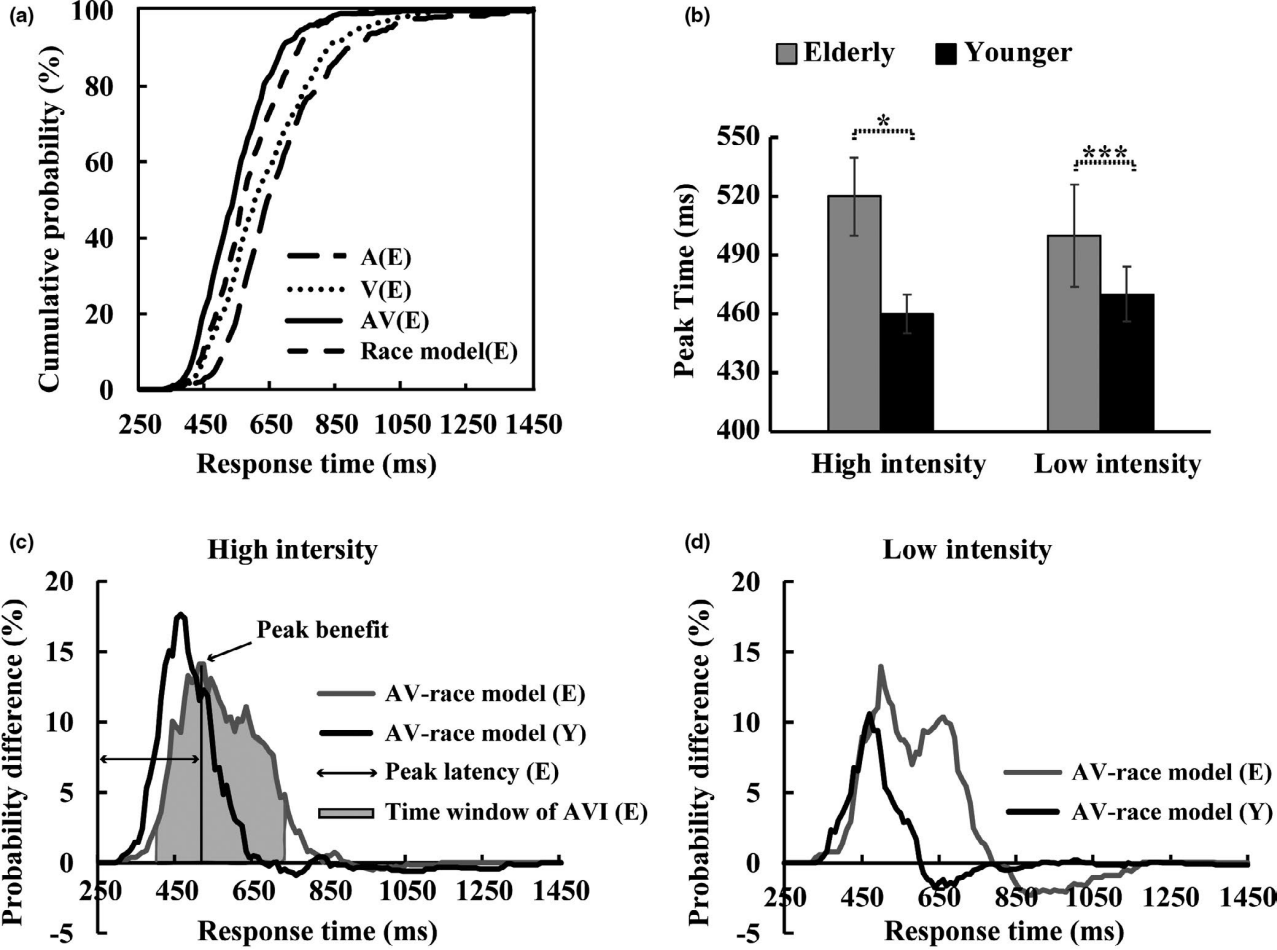
Probability difference of the bimodal audio–visual performance relative to the predicted race model. (a) CDFs for the response times to the auditory, visual, audio–visual stimuli, and the race models for the elderly adults under the high‐intensity‐stimulus condition. (b) Delayed AVI for the elderly adults in both the high‐ and low‐intensity‐stimulus sessions. A greater AUC was observed for the elderly adults under both (c) the high‐ and (d) the low‐intensity conditions than for younger adults. The error bar was also presented. ^*^ *p* ≤ .05, ^***^ *p* ≤ .001. E, elderly adults; Y, younger adults

Two‐tailed *t tests* were conducted between the audio–visual CDFs and the race model to evaluate the redundant nature effect in each 10‐ms time bin for each group under each condition (Figure 3a). The results showed that AVI occurred under all conditions (all *p* < .05) (Figure 3c, 3d and Table 1). The 2_group_ (Elderly, Younger) × 2_stimulus intensity_ (High, Low) for the positive AUC showed a significant main effect for group [*F*(1, 36) = 2.168, *p* = .015, *η*_p_^2^ = 0.571] and stimulus intensity [*F*(1, 36) = 5.629, *p* = .023, *η*_p_^2^ = 0.135], showing a higher AUC for elderly adults than for younger adults and a higher AUC under the high‐intensity‐stimulus condition than under the low‐intensity‐stimulus condition. However, no significant interaction between the group and the stimulus intensity [*F*(1, 36) = 0.355, *p* = .555, *η*_p_^2^ = 0.010] was found. These results indicated a much higher AVI ability in elderly adults compared with the younger adults under both the high‐intensity‐stimulus (3.5 versus. 2.7, *p* = .024) and low‐intensity‐stimulus (3.0 versus. 1.2, *p* = .005) conditions. In addition, the peak latency was significantly delayed for the elderly adults compared to the younger adults under both the high‐intensity‐stimulus (520 ms versus. 460 ms, *p* = .029) and the low‐intensity‐stimulus (510 ms versus. 470 ms, *p* = .0042) conditions (Figure 3b, Table 1). In addition, the time window for AVI was also delayed but widened in the elderly adults under both the high‐ and low‐intensity‐stimulus conditions (Table 1). Both the peak latency and integration time window illustrated that the AVI was delayed in the elderly adults.

**TABLE 1:** The time window, the peak latency, and the AUC of AVI for each stimulus type

	Time window (ms)	Peak latency(ms)	AUC
High intensity			
Elderly	390–690	520	3.5
Younger	360–560	460	2.7
Low intensity			
Elderly	460–690	510	3.0
Younger	430–500	470	1.2

### 3.2 EEG results

#### 3.2.1 Time course of mean PLI

**FIGURE 4:**
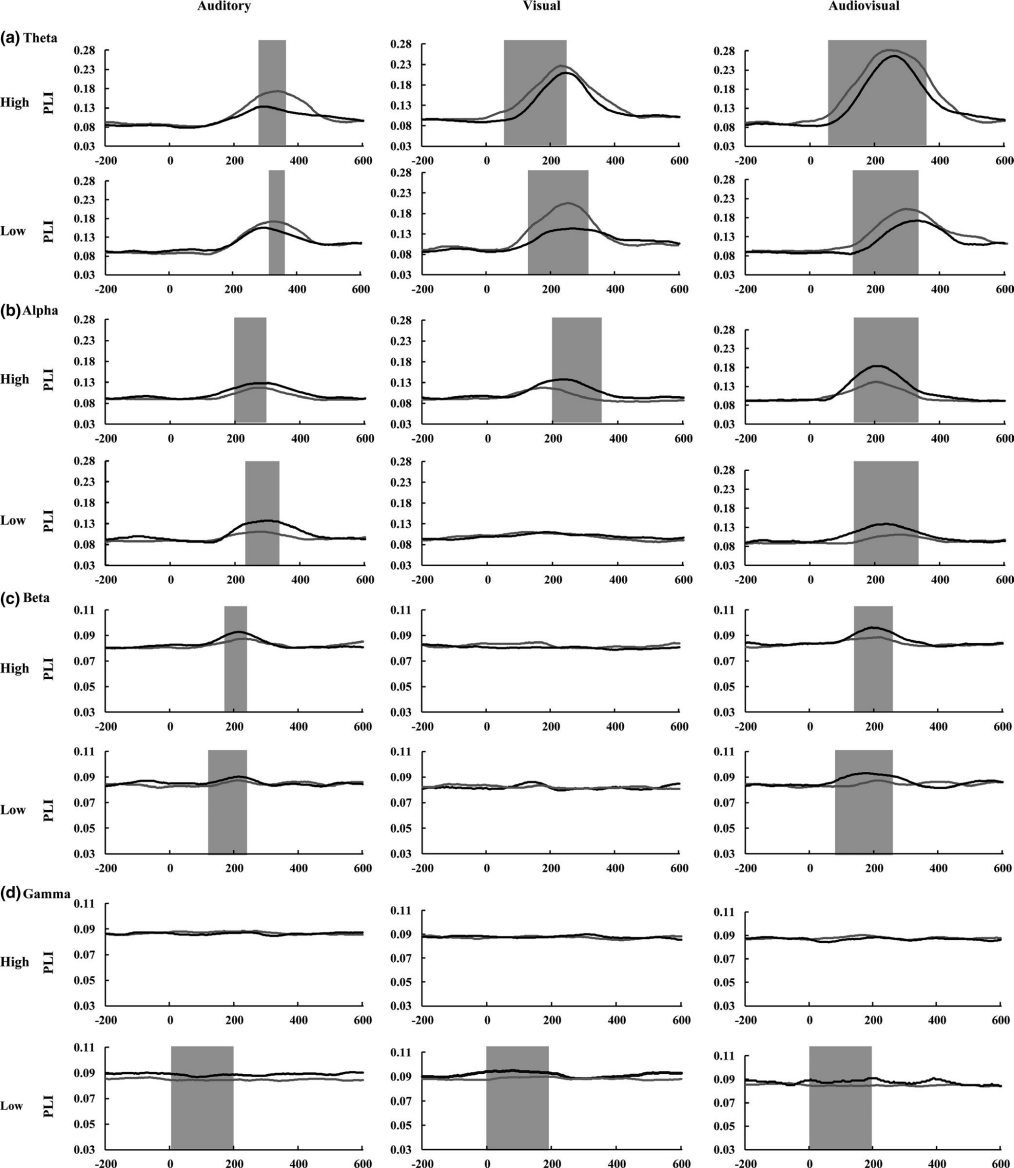
Time courses for the mean PLI for the elderly (gray) and younger (black) adults in (a) the theta bands with significantly different time courses marked with a gray background. (b) alpha bands, (c) beta bands, and (d) gamma bands

The one‐tailed *t* test for the elderly and younger adults was conducted for each stimulus condition, and the results showed that for the theta, alpha, and gamma bands, the time range was wider for the audio–visual stimuli than for the visual and auditory stimuli; however, there was no difference between the bimodal and unimodal stimuli for the gamma band (Table 2). To further investigate the main effects and interactions, the time course covering all of the variant time ranges was selected. Therefore, the ranges of 52–348 ms for the theta band (Figure 4a), 108–328 ms for the alpha band (Figure 4b), 108–240 ms for the beta band (Figure 4c), and 0–200 ms for the gamma band (Figure 4d) were obtained and averaged for further global functional connectivity analysis to assess global network strength.

**TABLE 2:** The time courses for the significant differences between the elderly and younger adults

	Stimulus intensity	A (ms)	V (ms)	AV (ms)
Theta	High	272–348	52–252	52–348
	Low	308–348	116–316	116–324
Alpha	High	200–284	200–328	108–328
	Low	248–328	–	108–328
Beta	High	172–236	–	332–240
	Low	112–236	–	108–240
Gamma	High	–	–	–
	Low	0–200	0–200	0–200

#### 3.2.2 Functional connectivity

**FIGURE 5:**
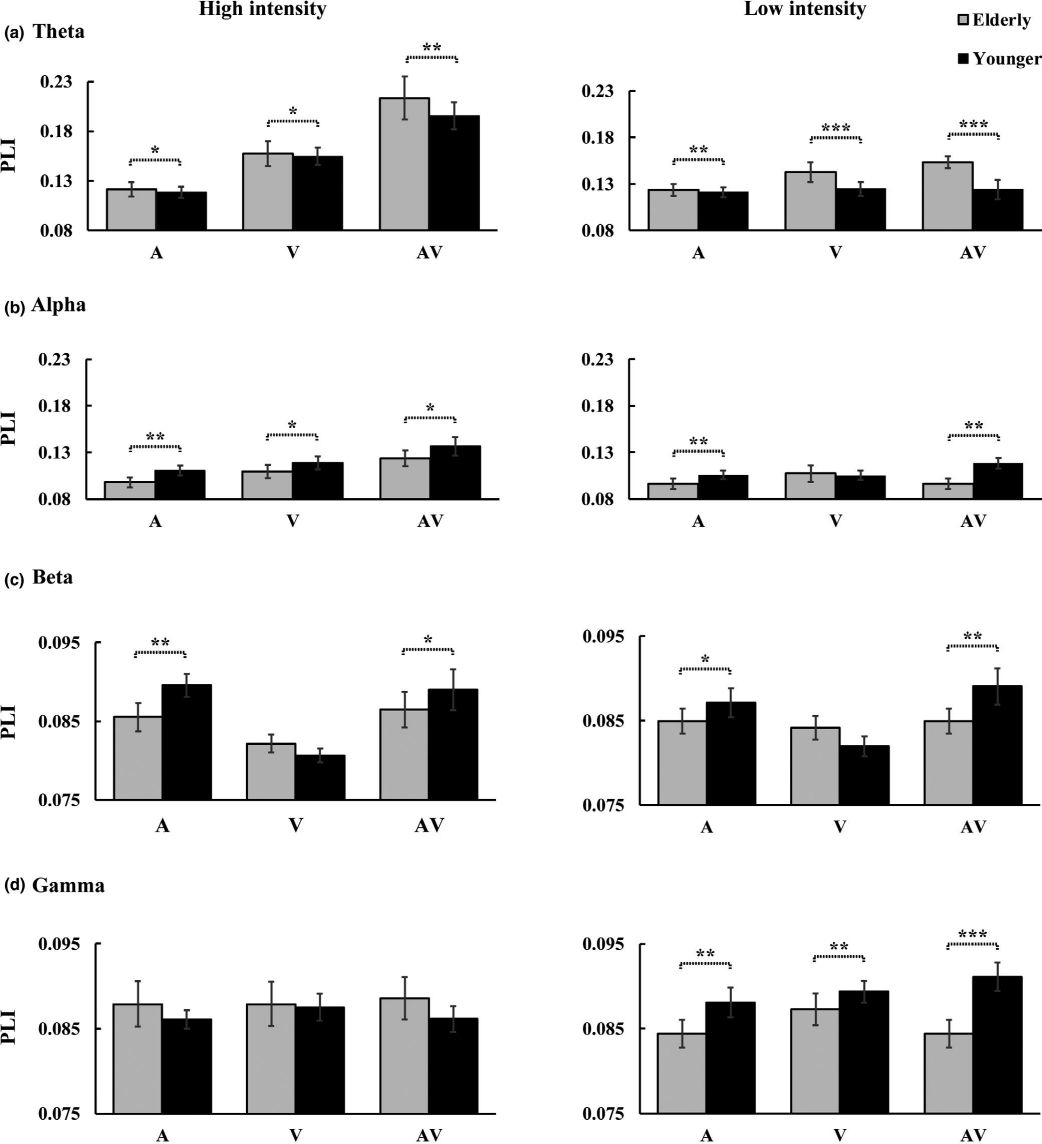
Comparison of the mean PLI values between the elderly and younger adults for the three stimulus types (A, V, AV) under both the high‐ and low‐intensity‐stimulus conditions for the theta bands (a), alpha bands (b), beta bands (c), and gamma bands (d). The standard error of mean (*SEM*) was also presented. ^*^ *p* ≤ .05, ^**^ *p* ≤ .01, ^***^ *p* ≤ .001

As in a previous study (Wang et al., 2017, 2018), the global functional connectivity was measured using the mean weights (PLI values) of connectivity. The mean PLI values were showed in Figure 5a for theta band, in Figure 5b for alpha band, in Figure 5c for beta band, and in Figure 5d for gamma band.

##### Theta band

2_group_(Elderly, Younger) × 2_stimulus intensity_ (High, Low) × 3_stimulus type_ (A, V, AV) ANOVA for theta band showed a significant stimulus intensity main effect [*F*(1, 36) = 23.786, *p* < .001, *η*_p_^2^ = 0.398], showing much stronger functional connectivity under the high‐intensity stimulus condition than under the low‐intensity stimulus condition, and stimulus type main effect [*F*(2, 72) = 31.500, *p* < .001, *η_p_^2^* = 0.398], showing the strongest functional connectivity during audio–visual stimulus processing and weakest functional connectivity during auditory stimulus processing (AV > A > V). Besides, there was significant main effect of group [*F*(1, 36) = 21.873, *p* < .001, *η*_p_^2^ = 0.962], indicating much stronger functional connectivity for the elderly adults than the younger adults. Additionally, a significant interaction between the stimulus intensity and the stimulus type [*F*(2, 72) = 21.829, *p* < .001, *η*_p_^2^ = 0.377] was also found. The paired comparison in the *post hoc* analysis showed that the functional connectivity was stronger under the high‐intensity‐stimulus condition than under the low‐intensity‐stimulus condition during audio–visual (*p <* .001) and auditory (*p* = .005) stimulus processing; however, there was no significant difference (*p* = .693) when processing the visual stimuli. Under the high‐intensity‐stimulus condition, the strongest functional connectivity was observed during audio–visual stimulus processing and the weakest during visual stimulus processing (AV > A > V, all *p* < .001). However, under the low‐intensity stimulus condition, there was no significant difference when processing the different stimuli (all *p *> .05). No other significant interactions were found (all *p* ≥ .270).

##### Alpha band

2_group_(Elderly, Younger) × 2_stimulus intensity_ (High, Low) × 3_stimulus type_ (A, V, AV) ANOVA for alpha band showed that similar functional connectivity with the theta band was found for the stimulus intensity [*F*(1, 36) = 4.748, *p* = .036, *η*_p_^2^ = 0.117] and the stimulus type [*F*(2, 72) = 9.154, *p* = .001 *η*_p_^2^ = 0.203]; however, the result for the group main effect [*F*(1, 36) = 2.607, *p* = .012, *η*_p_^2^ = 0.468] showed weaker functional connectivity for the elderly adults than the younger adults. In addition, a significant interaction between the stimulus intensity and the stimulus type [*F*(2, 72) = 7.377, *p* = .002, *η*_p_^2^ = 0.170] was also found. The paired comparison in the *post hoc* analysis showed that the functional connectivity was stronger under the high‐intensity‐stimulus condition than under the low‐intensity‐stimulus condition during audio–visual stimulus processing (*p =* .003); however, there was no significant difference when processing the auditory (*p =* .419) and visual (*p =* .182) stimuli. Under the high‐intensity‐stimulus condition, the functional connectivity was stronger during audio–visual stimulus processing than during auditory (*p =* .001) and visual (*p =* .002) stimuli processing. However, there was no significant difference during the processing of the auditory and visual stimuli (*p =* .213). Under the low‐intensity‐stimulus condition, no significant difference when processing the different stimuli was observed (all *p *> .05). No other significant interactions were found (all *p *> .05).

##### Beta band

2_group_(Elderly, Younger) × 2_stimulus intensity_ (High, Low) × 3_stimulus type_ (A, V, AV) ANOVA for beta band showed significant stimulus type main effect [*F*(2, 72) = 11.923, *p* < .001, *η*_p_^2^ = 0.249], showing that the strongest functional connectivity occurred during audio–visual stimulus processing, the weakest functional connectivity occurred during auditory stimulus processing (AV > A > V), and significant group main effect [*F*(1, 36) = 4.801, *p* = .038, *η*_p_^2^ = 0.322], showing that a much stronger functional connectivity was observed in the younger adults than in the elderly adults. In addition, a significant interaction between the stimulus intensity and the stimulus type [*F*(2, 72) = 3.490, *p* = .036, *η*_p_^2^ = 0.088] was also found. The *post hoc* analysis showed weakest functional connectivity during auditory stimulus processing than when processing the visual (all *p* ≤ .017) and audio–visual (all *p* ≤ .008); however, there was no significant difference between the visual and audio–visual stimuli processing (all *p* = 1.000) under both the high‐ and low‐intensity‐stimulus conditions. When processing all types of stimuli (A, V, and AV), there was no significant difference under the high‐ and low‐intensity conditions (all *p* ≥ .159). No other significant interactions or stimulus intensity main effects were found (all *p *> .05).

##### Gamma band

2_group_(Elderly, Younger) × 2_stimulus intensity_ (High, Low) × 3_stimulus type_ (A, V, AV) ANOVA for gamma band showed a group main effect [*F*(1, 36) = 1.259, *p* = .0269, *η*_p_^2^ = 0.234], showing that the younger adults exhibited much stronger functional connectivity than the elderly adults. Besides, the interaction between the stimulus intensity and group [*F*(1, 36) = 2.577, *p* = .0117, *η*_p_^2^ = 0.167] was also found, and the *post hoc* analysis showed a significant difference between the elderly and younger adults under the low‐intensity‐stimulus condition (*p* = .0045) but not under the high‐intensity‐stimulus condition. No other significant differences were found (all *p* ≥ .05).

## 4 DISCUSSION

Audio–visual integration was delayed under all conditions in elderly adults, and the delayed AVI was also found in the behavioral studies of Ren et al. (2016), and Wang et al. (2017, 2018), as well as the ERP results (Ren, Ren, et al., 2018; Wang et al., 2017, 2018). Colonius et al. proposed a “time‐window‐of‐integration model” and they presumed that cross‐modal information integration includes at least two serial stages of saccadic reaction times: an early afferent stage of peripheral processing (first stage) and a compound stage of converging sub‐processes (second stage) (Colonius & Diederich, 2004; Diederich et al., 2008). The first stage consists of very early sensory processing, and the processing time is assumed to be independent for unimodal sensory stimuli. If the peripheral processes in the first stage all terminate within a given time interval, multisensory integration is assumed to occur. Compared with the younger adults, the older adults showed a higher threshold for the perception of auditory and visual stimuli and a slower processing speed (Liu & Yan, 2007; Spear, 1993). Therefore, the delayed AVI might be mainly due to a unimodal functional decline, which results in slower signal processing. Besides, studies have demonstrated that the processing of higher‐intensity stimuli was faster than that of lower‐intensity stimuli (Glickfeld, Histed, & Maunsell, 2013; Parker & Salzen, 1982; Skottun, Bradley, Sclar, Ohzawa, & Freeman, 1987; Stone & Thompson, 1992); therefore, the delayed AVI under the low‐intensity stimulus condition might be due to slower signal processing. However, the elderly adults achieved a higher AVI effect and widened time window. In the current study, although all the responses were slower, the elderly adults completed the experiment as successfully as the younger adults, achieving a similar hit rate to the younger adults. Age‐related AVI study conducted by Ren ta al. also reported a slower response and a delayed later AVI wave, but the earliest integration in the occipital region (80–110 ms) occurred specifically in elderly adults (Ren et al., 2018). Besides, Wang et al. (2017, 2018) reported an increased functional connection and network efficiency for the elderly adults even the slower response to all stimuli. Therefore, the enhanced AVI effect and widened time window might be the compensatory mechanism of the general functional decline in aging brain.

Increased theta‐ and reduced alpha‐band functional connectivity were found in the elderly adults. Extensive studies have yielded evidence of attention decline in elderly adults (Kok, 2000; Plude et al., 1994; Quigley et al., 2010), and it becomes more difficult to complete many cognitive tasks with normal aging, such as discriminate simultaneity and temporal order among stimuli tasks, leading to an increase in the width of the temporal binding window compared to that of younger adults (Bedard & Barnett‐Cowan, 2016; Diederich & Colonius, 2015; Poliakoff, Shore, Lowe, & Spence, 2006; Setti, Burke, et al., 2011; Setti, Finnigan, et al., 2011). However, the elderly adults still maintained the ability to complete a number of cognitive tasks as younger adults. Considering that attention is the key factor for cognitive performance, we propose that elderly adults should allocate more attentional recourse than younger adults to perform the same task. Attention influences AVI in multiple stages and that the AVI effect is stronger under attended conditions than under unattended conditions (Talsma, Doty, & Woldorff, 2007; Talsma, Senkowski, Soto‐Faraco, & Woldorff, 2010; Talsma, Senkowski, & Woldorff, 2009; Talsma & Woldorff, 2005); therefore, elderly adults exhibit stronger AVI ability behaviorally than younger adults. Furthermore, theta oscillation is associated with attention (Keller et al., 2017); therefore, the enhanced theta‐band functional connection might be a reflection of higher occupation of attentional recourse in elderly adults. This finding was consistent with conclusion of Wang et al.’s studies, who proposed that the increased functional connectivity in elderly adults was mainly due to higher cognitive demand (Wang et al., 2017, 2018); however, we specifically proposed that the increased AVI might be attributed to more attentional resource occupation. Additionally, the current study is the unmatched design between target and nontarget trials, which will lead to oddball effect. The oddball effect is very widely seen in terms ERPs like P300's, and may introduce biases cross‐frequency, which might affect phase‐lagged indices. According to Lavie et al.'s study, the attentional recourse for one person is limited (Lavie & Tsal, 1994). When multiple tasks are conducted simultaneously, if one task uses more attentional resources, the other tasks will be allocated relatively less attention. The elderly adults allocated more attentional resources in the hand‐held tool recognition task than younger adult, leading fewer attentional resources are left to resist the task‐irrelevant distractor in elderly adults than in younger adults (Lavie & Tsal, 1994). The activity of the alpha band has been recognized as a marker of intentional ignoring (Friese et al., 2016; Keller et al., 2017; Yordanova et al., 1998); therefore, it is reasonable that the elderly adults exhibit a reduced alpha‐band functional connection.

Additionally, reduced beta‐ and gamma‐band functional connectivities were found in elderly adults. Although numerous studies have investigated the association between beta oscillation and sensory motor function, Sakowitz et al. (2005) firstly reported that the beta activity play an important role in response time facilitation for audio–visual stimuli. To further clarify the relationship between audio–visual interplay in the beta frequency range and motor processing, Senkowski et al. (2006) conducted a stimulus detection experiment, during which a fast response was instructed when random auditory, visual, or audio–visual stimuli were presented. Their results found a negative association between the beta activity and response time over all the stimulus types, which suggest that beta activity is directly linked to audio–visual response time facilitation. In the current study, all the responses to the bimodal audio–visual stimuli were faster than the unimodal auditory and visual stimuli, and all the responses of the elderly adults were significantly slower than younger adults. Therefore, we proposed that the reduced beta‐band functional connection for elderly adults might be mainly due to the slower sensory processing in the latter stage. Besides, the procedure of hand‐held tool recognition contains episodic memory and flexible response. However, aging effect studies have yielded evidence of dysfunction in executive function (Cepeda et al., 2001; Kramer et al., 1999), episodic memory (Loftus, 1984), and overall flexibility (Grady, 2012). Therefore, the reduced gamma‐band functional connectivity might be mainly due to general cognitive functional decline.

Furthermore, consistent with our hypothesis that the IE effect was not occurred, showing that the AVI effect was lower under the low‐intensity stimulus condition for both the elderly and younger adults, as well as the functional connectivity of frequency bands. This finding together with previous studies (Stein & Meredith, 1993; Stevenson & James, 2009) suggested that it might be the certain brain regions exhibit IE effects but the whole brain does not. In the current study, the global functional connectivity was used to evaluate the IE effect; therefore, it is reasonable that the IE effect was not observed. Besides, studies have shown that cross‐modal stimuli could attract much stronger and more stable attention than unimodal stimuli, making it difficult to be disturbed by other distractors (Santangelo & Spence, 2007). Given that the theta band is an index of attention and that the alpha band is an index of suppressing distractors, it is reasonable that significantly higher theta‐band and alpha‐band functional connectivity were elicited by the audio–visual stimuli. In addition, in the current study, all of the stimuli were presented randomly without any prior indication, and the stimulus with salient features could elicit higher vigilance during the experiment, tolerating the external distractors (Petersen & Posner, 2012). Therefore, the theta‐band and alpha‐band functional connectivity were higher under the high‐intensity‐stimulus condition than under the low‐intensity‐stimulus condition.

## 5 CONCLUSION

Although perceptual deficits in elderly adults have been reported extensively, our findings confirmed that elderly adults maximize their performance by enhancing audio–visual integration and strengthening theta‐band functional brain connectivity when processing dynamic audio–visual hand‐held tool stimuli. The results further demonstrated that in the case of sensory processing, the theta, alpha, beta, and gamma activity might participate in different stages of perception.

## CONFLICT OF INTEREST

The authors declare that they have no competing interests.

## AUTHOR CONTRIBUTION

Yanna Ren and Weiping Yang conceived and designed the experiments. Ao Guo and Tao Wang collected the data. Yanna Ren analyzed the data, wrote the draft manuscript, and received comments from Weiping Yang.

### Peer Review

The peer review history for this article is available at https://publons.com/publon/10.1002/brb3.1759.

## Supporting information

Supinfo

## Data Availability

The data that support the findings of this study are available from the corresponding author upon reasonable request.
