# Supplementary material for: Age‐related functional brain connectivity during audio–visual hand‐held tool recognition
Source: Brain Behav. 2020 Jul 18;10(9):e01759. doi: 10.1002/brb3.1759 (PMC7507049; doi:10.1002/brb3.1759)
Supplement: Supplementary file 1 — Supinfo [file BRB3-10-e01759-s001.docx]

**Supporting Information**

According to previous studies, five regions of interests (ROIs) (frontal: F7, F3, Fz, F4, and F8; fronto-central: FC5, FC1, FC2, and FC6; central: C3, Cz, and C4; centro-parietal: CP5, CP1, CP2, and CP6; and occipital: O1, O2, and Oz) in the 0 – 600 ms time interval and the 1 – 50 Hz frequency range were selected. According to the *one-way* ANOVA for each ROI, there was no significant lateralization effect; therefore, we chose one electrode in each ROI (Fz, FC1, Cz, CP1 and Oz), and the power spectrum map for the five selected electrodes in all stimulus types is shown in Figure S1 for high stimulus-intensity condition, and Figure S2 for low stimulus-intensity condition.


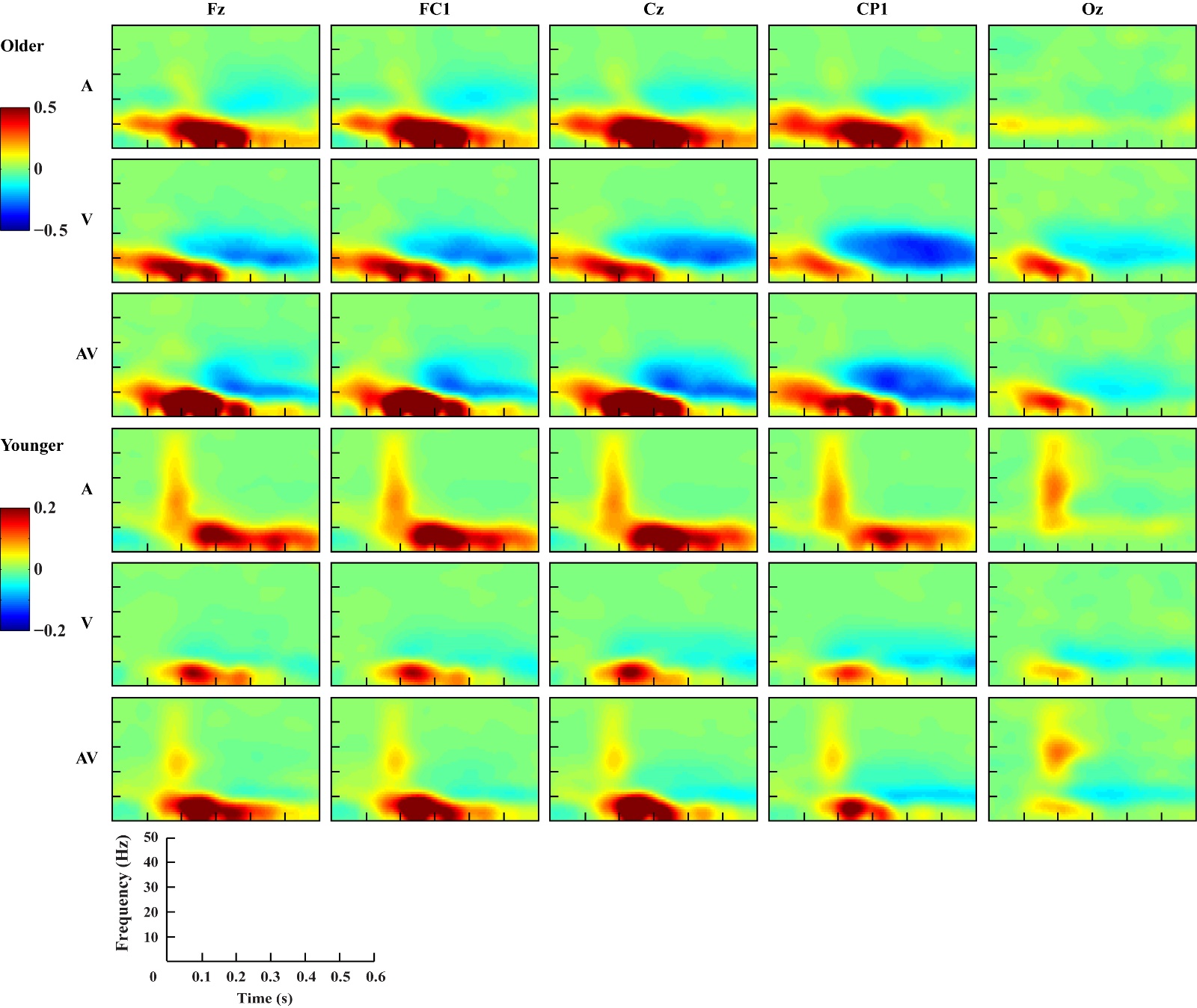


Figure S1 Spectral power of older and younger adults for a time interval of 0 – 600 ms for visual, auditory, and audio-visual stimuli in ROIs for high-stimulus-intensity condition.


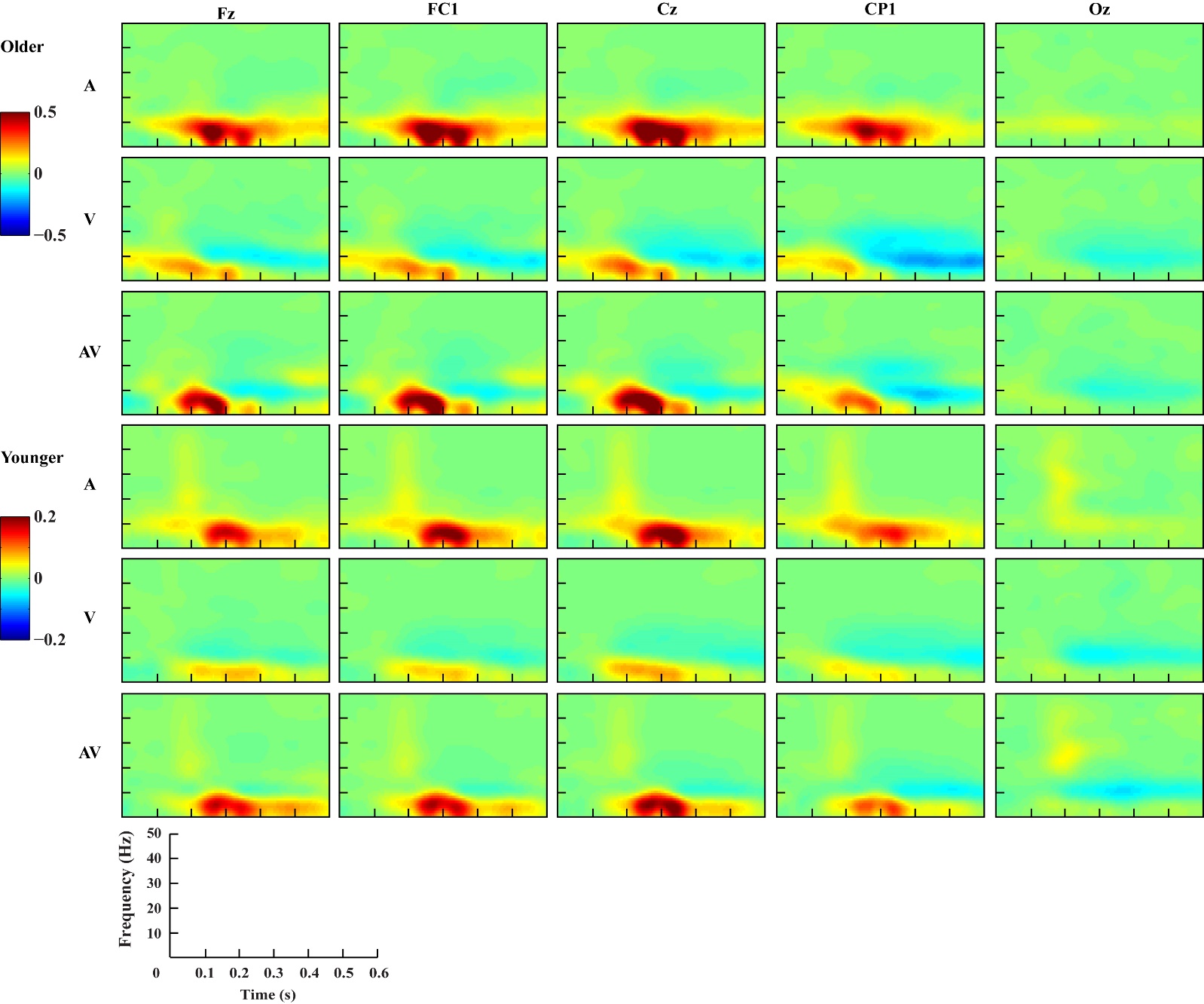


Figure S2 Spectral power of older and younger adults for a time interval of 0 – 600 ms for visual, auditory, and audio-visual stimuli in ROIs for low-stimulus-intensity condition.
